# Potassium zinc borate, KZnB_3_O_6_

**DOI:** 10.1107/S1600536810015175

**Published:** 2010-04-30

**Authors:** Yang Wu, Ji-Yong Yao, Jian-Xiu Zhang, Pei-Zhen Fu, Yi-Cheng Wu

**Affiliations:** a Key Laboratory of Functional Crystal and Laser Technology, Technical Institute of Physics and Chemistry, Chinese Academy of Sciences, Beijing 100190, People’s Republic of China

## Abstract

The title compound, KZnB_3_O_6_ contains a remarkable [B_6_O_12_]^6−^ group (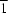 symmetry) formed by two rings linked by edge-sharing BO_4_ tetra­hedra, a feature that has only been observed previously under high pressure conditions. These borate groups are connected through distorted ZnO_4_ tetra­hedra in edge-shared pairs (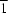 symmetry), forming a three-dimensional network whose cavities are filled by K^+^ cations.

## Related literature

For an independent determination of the title compound, see: Chen *et al.* (2010 ▶). For related structures, see: Chen *et al.* (2005 ▶); Emme & Huppertz (2003 ▶, 2004 ▶, 2005 ▶); Huppertz (2003 ▶); Huppertz & Emme (2004 ▶); Huppertz & von der Eltz (2002 ▶); Knyrim *et al.* (2007 ▶); Smith *et al.* (1992 ▶).

## Experimental

### 

#### Crystal data

KZnB_3_O_6_
*M* *_r_* = 232.90
Triclinic, 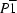

*a* = 6.7139 (13) Å
*b* = 6.9301 (14) Å
*c* = 7.0632 (14) Å
α = 63.12 (3)°
β = 72.02 (3)°
γ = 68.99 (3)°
*V* = 269.37 (12) Å^3^
*Z* = 2
Mo *K*α radiation
μ = 5.29 mm^−1^
*T* = 93 K
0.50 × 0.30 × 0.20 mm

#### Data collection

Rigaku Saturn 724+ diffractometer
Absorption correction: multi-scan (*ABSCOR*; Higashi, 1995 ▶) *T*_min_ = 0.480, *T*_max_ = 1.000
2707 measured reflections
1223 independent reflections
1118 reflections with *I* > 2σ(*I*)
*R*_int_ = 0.023

#### Refinement

*R*[*F* ^2^ > 2σ(*F* ^2^)] = 0.020
*wR*(*F* ^2^) = 0.050
*S* = 0.94
1223 reflections
100 parameters
Δρ_max_ = 0.38 e Å^−3^
Δρ_min_ = −0.58 e Å^−3^

### 

Data collection: *CrystalClear* (Rigaku, 2008 ▶); cell refinement: *CrystalClear*; data reduction: *CrystalClear*; program(s) used to solve structure: *SHELXS97* (Sheldrick, 2008 ▶); program(s) used to refine structure: *SHELXL97* (Sheldrick, 2008 ▶); molecular graphics: *ATOMS* (Dowty, 1998 ▶); software used to prepare material for publication: *SHELXL97*.

## Supplementary Material

Crystal structure: contains datablocks I, global. DOI: 10.1107/S1600536810015175/mg2092sup1.cif

Structure factors: contains datablocks I. DOI: 10.1107/S1600536810015175/mg2092Isup2.hkl

Additional supplementary materials:  crystallographic information; 3D view; checkCIF report

## Figures and Tables

**Fig. 1.**
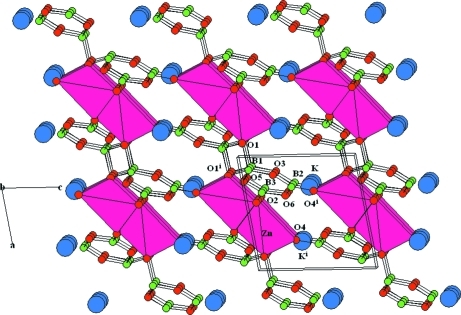
KZnB3O6 viewed along b axis showing ZnO4 tetrahedra (magenta) linked by BO3 triangles and BO4 tetrahedra, with K+ cations (blue) located within the three-dimensional framework.

**Fig. 2.**
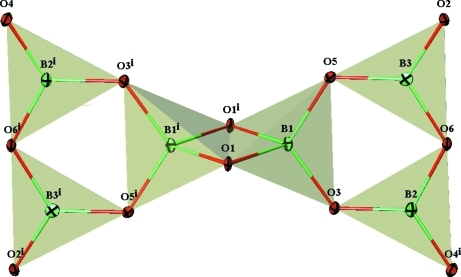
[B6O12]6- anion, with displacement ellipsoids drawn at the 50% probability level. [Symmetry code: (i) -x, -y, -z.]

## supplementary crystallographic information

### Comment

In efforts to identify new borates as optical materials or catalysts, investigations have been carried out in the K_2_O–ZnO–B_2_O_3_ system, where only one quaternary compound so far, KZn_4_B_3_O_9_, has been structurally characterized (Smith *et al.*, 1992; Chen *et al.*, 2005). Here, we report a new compound, KZnB_3_O_6_, with an unusual anion group. It is the first example of a borate prepared at ambient conditions that contains edge-sharing BO_4_ tetrahedra, a feature that has only been previously found in high-pressure phases (Huppertz & von der Eltz, 2002; Huppertz, 2003; Huppertz & Emme, 2004; Emme & Huppertz, 2003, 2004, 2005; Knyrim *et al.*, 2007).

The structure consists of BO_3_ triangles, BO_4_ tetrahedra, and ZnO_4_ tetrahedra linked to form a three-dimensional framework whose cavities are filled with K^+^ cations in nine-coordinate environments (Fig. 1). The fundamental building block is a [B_6_O_12_]^6-^ anion in which two six-membered rings formed by borate polyhedra are connected through a shared edge between two BO_4_ tetrahedra (Fig. 2). Within the resulting B_2_O_2_ ring, the transannular B–B distance [2.080 (5) Å] is similar to those in HP-NiB_2_O_4_ [2.088 (2) Å] and Dy_4_B_6_O_15_ [2.072 (8) Å] (Knyrim *et al.*, 2007; Huppertz & von der Eltz, 2002). The Raman spectrum of KZnB_3_O_6_ shows bands at 1319 and 1456 cm^-1^, which lie in the range (about 1200 to 1450 cm^-1^) expected for the Raman-active modes of edge-sharing BO_4_ tetrahedra (Knyrim *et al.*, 2007). Two distorted ZnO_4_ tetrahedra also share a common edge, similar to the case of Zn_3_B_2_O_6_ (Chen *et al.*, 2005).

### Experimental

A mixture of 7 mmol K_2_CO_3_, 10 mmol ZnO, and 43 mmol H_3_BO_3_ (all from Beijing Chemical Reagents Company) was heated to 1173 K in a platinum crucible. The transparent melt was cooled slowly from 1173 K to 1053 K at 1 K h^-1^. Upon further cooling to room temperature, column-shaped colorless crystals were obtained.

### Refinement

(type here to add refinement details)

### Crystal data

**Table:**

KZnB_3_O_6_	*Z* = 2
*M**_r_* = 232.90	*F*(000) = 224
Triclinic, *P*1	*D*_x_ = 2.871 Mg m^−^^3^
Hall symbol: -P 1	Mo *K*α radiation, λ = 0.71073 Å
*a* = 6.7139 (13) Å	Cell parameters from 1009 reflections
*b* = 6.9301 (14) Å	θ = 3.3–27.5°
*c* = 7.0632 (14) Å	µ = 5.29 mm^−^^1^
α = 63.12 (3)°	*T* = 93 K
β = 72.02 (3)°	Prism, colorless
γ = 68.99 (3)°	0.50 × 0.30 × 0.20 mm
*V* = 269.37 (12) Å^3^	

### Data collection

**Table:**

Rigaku Saturn 724+ diffractometer	1223 independent reflections
Radiation source: fine-focus sealed tube	1118 reflections with *I* > 2σ(*I*)
graphite	*R*_int_ = 0.023
ω scans	θ_max_ = 27.5°, θ_min_ = 3.3°
Absorption correction: multi-scan (*ABSCOR*; Higashi, 1995)	*h* = −7→8
*T*_min_ = 0.480, *T*_max_ = 1.000	*k* = −8→9
2707 measured reflections	*l* = −9→8

### Refinement

**Table:**

Refinement on *F*^2^	0 restraints
Least-squares matrix: full	Primary atom site location: structure-invariant direct methods
*R*[*F*^2^ > 2σ(*F*^2^)] = 0.020	Secondary atom site location: difference Fourier map
*wR*(*F*^2^) = 0.050	*w* = 1/[σ^2^(*F*_o_^2^) + (0.030*P*)^2^] where *P* = (*F*_o_^2^ + 2*F*_c_^2^)/3
*S* = 0.94	(Δ/σ)_max_ = 0.001
1223 reflections	Δρ_max_ = 0.38 e Å^−^^3^
100 parameters	Δρ_min_ = −0.58 e Å^−^^3^

### Special details

**Table:**

Geometry. All esds (except the esd in the dihedral angle between two l.s. planes) are estimated using the full covariance matrix. The cell esds are taken into account individually in the estimation of esds in distances, angles and torsion angles; correlations between esds in cell parameters are only used when they are defined by crystal symmetry. An approximate (isotropic) treatment of cell esds is used for estimating esds involving l.s. planes.
Refinement. Refinement of *F*^2^ against ALL reflections. The weighted *R*-factor wR and goodness of fit *S* are based on *F*^2^, conventional *R*-factors *R* are based on *F*, with *F* set to zero for negative *F*^2^. The threshold expression of *F*^2^ > σ(*F*^2^) is used only for calculating *R*-factors(gt) etc. and is not relevant to the choice of reflections for refinement. *R*-factors based on *F*^2^ are statistically about twice as large as those based on *F*, and *R*- factors based on ALL data will be even larger.

### Fractional atomic coordinates and isotropic or equivalent isotropic displacement parameters (Å^2^)

**Table:**

	*x*	*y*	*z*	*U*_iso_*/*U*_eq_	
K	0.25708 (8)	0.29494 (8)	0.57923 (7)	0.00606 (12)	
Zn	0.67490 (4)	0.35335 (4)	0.12712 (4)	0.00346 (9)	
B1	0.0964 (4)	0.9933 (4)	0.0944 (4)	0.0048 (5)	
B2	0.2441 (4)	0.8171 (4)	0.4350 (4)	0.0050 (5)	
B3	0.2977 (4)	0.1817 (4)	0.1685 (4)	0.0052 (5)	
O1	0.8584 (2)	0.0829 (3)	0.0849 (2)	0.0046 (3)	
O2	0.3702 (3)	0.3605 (3)	0.1211 (2)	0.0054 (3)	
O3	0.1393 (3)	0.8112 (3)	0.2984 (2)	0.0055 (3)	
O4	0.7213 (3)	0.3533 (3)	0.3794 (2)	0.0065 (3)	
O5	0.1989 (3)	0.1716 (3)	0.0323 (2)	0.0056 (3)	
O6	0.3232 (3)	0.0056 (3)	0.3678 (2)	0.0058 (3)	

### Atomic displacement parameters (Å^2^)

**Table:**

	*U*^11^	*U*^22^	*U*^33^	*U*^12^	*U*^13^	*U*^23^
K	0.0064 (3)	0.0064 (2)	0.0060 (2)	−0.00214 (19)	−0.00063 (19)	−0.00281 (19)
Zn	0.00389 (15)	0.00353 (14)	0.00324 (14)	−0.00047 (10)	−0.00134 (10)	−0.00143 (10)
B1	0.0042 (12)	0.0045 (12)	0.0061 (11)	0.0002 (10)	−0.0021 (9)	−0.0027 (9)
B2	0.0038 (12)	0.0070 (12)	0.0047 (11)	−0.0002 (10)	−0.0002 (9)	−0.0041 (9)
B3	0.0027 (12)	0.0052 (12)	0.0061 (11)	0.0011 (10)	−0.0001 (9)	−0.0030 (9)
O1	0.0036 (8)	0.0068 (8)	0.0046 (7)	−0.0004 (6)	−0.0018 (6)	−0.0032 (6)
O2	0.0048 (8)	0.0056 (8)	0.0062 (7)	−0.0020 (6)	−0.0024 (6)	−0.0013 (6)
O3	0.0058 (8)	0.0053 (8)	0.0055 (7)	−0.0013 (6)	−0.0030 (6)	−0.0008 (6)
O4	0.0096 (9)	0.0056 (8)	0.0050 (7)	−0.0022 (7)	−0.0034 (6)	−0.0013 (6)
O5	0.0074 (8)	0.0052 (7)	0.0044 (7)	−0.0024 (6)	−0.0019 (6)	−0.0008 (6)
O6	0.0071 (8)	0.0059 (8)	0.0056 (7)	−0.0022 (7)	−0.0035 (6)	−0.0013 (6)

### Geometric parameters (Å, °)

**Table:**

K—O4^i^	2.6404 (16)	B1—B1^viii^	2.080 (5)
K—O3^ii^	2.7890 (18)	B1—K^ii^	3.286 (3)
K—O1^iii^	2.791 (2)	B1—K^ix^	3.330 (3)
K—O6	2.8476 (17)	B2—O4^i^	1.326 (3)
K—O5^iv^	2.8491 (17)	B2—O3	1.380 (3)
K—O6^iii^	2.856 (2)	B2—O6^vi^	1.412 (3)
K—O2	2.9418 (17)	B2—K^i^	3.135 (3)
K—O4	3.0888 (19)	B3—O2	1.357 (3)
K—B2^i^	3.135 (3)	B3—O5	1.363 (3)
K—O3	3.144 (2)	B3—O6	1.395 (3)
K—B3	3.244 (3)	O1—B1^v^	1.492 (3)
K—B2	3.261 (3)	O1—B1^x^	1.503 (3)
Zn—O4	1.9013 (15)	O1—K^iii^	2.791 (2)
Zn—O1	1.9347 (17)	O2—Zn^v^	1.9628 (18)
Zn—O2^v^	1.9628 (18)	O3—K^ii^	2.7890 (18)
Zn—O2	2.0414 (16)	O4—B2^i^	1.326 (3)
Zn—Zn^v^	2.9584 (14)	O4—K^i^	2.6404 (16)
B1—O3	1.450 (3)	O5—B1^xi^	1.458 (3)
B1—O5^vi^	1.458 (3)	O5—K^xii^	2.8491 (17)
B1—O1^v^	1.492 (3)	O6—B2^xi^	1.412 (3)
B1—O1^vii^	1.503 (3)	O6—K^iii^	2.856 (2)
			
O4^i^—K—O3^ii^	119.60 (6)	O1—Zn—K^iii^	41.67 (5)
O4^i^—K—O1^iii^	125.80 (5)	O2^v^—Zn—K^iii^	153.56 (5)
O3^ii^—K—O1^iii^	52.15 (6)	O2—Zn—K^iii^	83.72 (6)
O4^i^—K—O6	156.86 (5)	Zn^v^—Zn—K^iii^	121.23 (3)
O3^ii^—K—O6	76.47 (5)	K—Zn—K^iii^	75.71 (4)
O1^iii^—K—O6	76.88 (5)	O4—Zn—K^i^	32.09 (5)
O4^i^—K—O5^iv^	75.13 (5)	O1—Zn—K^i^	131.51 (5)
O3^ii^—K—O5^iv^	81.16 (6)	O2^v^—Zn—K^i^	85.63 (5)
O1^iii^—K—O5^iv^	51.22 (5)	O2—Zn—K^i^	118.91 (5)
O6—K—O5^iv^	126.26 (5)	Zn^v^—Zn—K^i^	106.44 (3)
O4^i^—K—O6^iii^	103.77 (5)	K—Zn—K^i^	75.22 (3)
O3^ii^—K—O6^iii^	127.90 (5)	K^iii^—Zn—K^i^	120.74 (2)
O1^iii^—K—O6^iii^	79.99 (6)	O3—B1—O5^vi^	113.83 (19)
O6—K—O6^iii^	73.35 (6)	O3—B1—O1^v^	113.47 (18)
O5^iv^—K—O6^iii^	83.76 (6)	O5^vi^—B1—O1^v^	111.49 (18)
O4^i^—K—O2	109.74 (5)	O3—B1—O1^vii^	112.28 (18)
O3^ii^—K—O2	101.75 (6)	O5^vi^—B1—O1^vii^	111.87 (19)
O1^iii^—K—O2	124.46 (5)	O1^v^—B1—O1^vii^	92.02 (17)
O6—K—O2	47.88 (5)	O3—B1—B1^viii^	124.0 (2)
O5^iv^—K—O2	171.41 (5)	O5^vi^—B1—B1^viii^	122.1 (2)
O6^iii^—K—O2	88.14 (6)	O1^v^—B1—B1^viii^	46.22 (12)
O4^i^—K—O4	72.24 (6)	O1^vii^—B1—B1^viii^	45.79 (12)
O3^ii^—K—O4	167.20 (5)	O3—B1—K^ii^	57.50 (11)
O1^iii^—K—O4	126.59 (6)	O5^vi^—B1—K^ii^	150.31 (15)
O6—K—O4	90.79 (5)	O1^v^—B1—K^ii^	97.21 (12)
O5^iv^—K—O4	107.95 (5)	O1^vii^—B1—K^ii^	57.78 (10)
O6^iii^—K—O4	46.94 (5)	B1^viii^—B1—K^ii^	72.83 (13)
O2—K—O4	67.96 (5)	O3—B1—K^ix^	151.61 (16)
O4^i^—K—B2^i^	79.31 (7)	O5^vi^—B1—K^ix^	58.34 (11)
O3^ii^—K—B2^i^	154.09 (6)	O1^v^—B1—K^ix^	56.22 (10)
O1^iii^—K—B2^i^	103.00 (7)	O1^vii^—B1—K^ix^	95.19 (13)
O6—K—B2^i^	91.80 (6)	B1^viii^—B1—K^ix^	70.52 (13)
O5^iv^—K—B2^i^	87.75 (7)	K^ii^—B1—K^ix^	143.35 (9)
O6^iii^—K—B2^i^	26.75 (5)	O4^i^—B2—O3	121.2 (2)
O2—K—B2^i^	86.28 (7)	O4^i^—B2—O6^vi^	120.5 (2)
O4—K—B2^i^	24.59 (6)	O3—B2—O6^vi^	118.2 (2)
O4^i^—K—O3	47.12 (4)	O4^i^—B2—K^i^	75.76 (14)
O3^ii^—K—O3	102.99 (5)	O3—B2—K^i^	128.91 (16)
O1^iii^—K—O3	150.43 (5)	O6^vi^—B2—K^i^	65.55 (12)
O6—K—O3	116.19 (5)	O4^i^—B2—K	51.19 (11)
O5^iv^—K—O3	116.19 (5)	O3—B2—K	72.85 (12)
O6^iii^—K—O3	128.27 (5)	O6^vi^—B2—K	158.31 (17)
O2—K—O3	71.27 (5)	K^i^—B2—K	92.88 (8)
O4—K—O3	81.38 (6)	O2—B3—O5	122.7 (2)
B2^i^—K—O3	102.92 (7)	O2—B3—O6	117.3 (2)
O4^i^—K—B3	133.81 (6)	O5—B3—O6	119.95 (19)
O3^ii^—K—B3	82.79 (6)	O2—B3—K	65.06 (11)
O1^iii^—K—B3	100.12 (6)	O5—B3—K	149.00 (16)
O6—K—B3	25.42 (5)	O6—B3—K	61.19 (11)
O5^iv^—K—B3	151.06 (6)	B1^v^—O1—B1^x^	87.98 (17)
O6^iii^—K—B3	87.36 (6)	B1^v^—O1—Zn	132.19 (14)
O2—K—B3	24.73 (5)	B1^x^—O1—Zn	125.03 (13)
O4—K—B3	85.14 (6)	B1^v^—O1—K^iii^	97.40 (12)
B2^i^—K—B3	96.54 (7)	B1^x^—O1—K^iii^	95.11 (12)
O3—K—B3	90.81 (6)	Zn—O1—K^iii^	110.89 (7)
O4^i^—K—B2	23.03 (5)	B3—O2—Zn^v^	126.73 (15)
O3^ii^—K—B2	117.32 (7)	B3—O2—Zn	126.50 (16)
O1^iii^—K—B2	145.28 (6)	Zn^v^—O2—Zn	95.24 (7)
O6—K—B2	136.67 (6)	B3—O2—K	90.21 (13)
O5^iv^—K—B2	97.00 (6)	Zn^v^—O2—K	126.87 (7)
O6^iii^—K—B2	113.85 (6)	Zn—O2—K	87.73 (6)
O2—K—B2	88.87 (6)	B2—O3—B1	123.45 (18)
O4—K—B2	71.34 (6)	B2—O3—K^ii^	124.93 (14)
B2^i^—K—B2	87.12 (8)	B1—O3—K^ii^	96.48 (13)
O3—K—B2	24.79 (5)	B2—O3—K	82.36 (13)
B3—K—B2	111.77 (7)	B1—O3—K	149.76 (13)
O4—Zn—O1	109.34 (7)	K^ii^—O3—K	77.01 (5)
O4—Zn—O2^v^	117.70 (7)	B2^i^—O4—Zn	128.77 (15)
O1—Zn—O2^v^	120.18 (7)	B2^i^—O4—K^i^	105.78 (13)
O4—Zn—O2	117.57 (7)	Zn—O4—K^i^	125.42 (8)
O1—Zn—O2	104.70 (7)	B2^i^—O4—K	79.65 (13)
O2^v^—Zn—O2	84.76 (7)	Zn—O4—K	86.04 (6)
O4—Zn—Zn^v^	128.89 (6)	K^i^—O4—K	107.76 (6)
O1—Zn—Zn^v^	120.57 (5)	B3—O5—B1^xi^	122.58 (18)
O2^v^—Zn—Zn^v^	43.41 (5)	B3—O5—K^xii^	136.44 (13)
O2—Zn—Zn^v^	41.35 (5)	B1^xi^—O5—K^xii^	95.84 (12)
O4—Zn—K	61.28 (5)	B3—O6—B2^xi^	121.34 (18)
O1—Zn—K	117.35 (6)	B3—O6—K	93.39 (12)
O2^v^—Zn—K	116.70 (6)	B2^xi^—O6—K	130.47 (13)
O2—Zn—K	56.78 (5)	B3—O6—K^iii^	118.51 (14)
Zn^v^—Zn—K	85.42 (3)	B2^xi^—O6—K^iii^	87.70 (14)
O4—Zn—K^iii^	88.71 (6)	K—O6—K^iii^	106.65 (6)
			
O4^i^—K—Zn—O4	50.46 (9)	K^iii^—Zn—O2—Zn^v^	−156.16 (5)
O3^ii^—K—Zn—O4	−170.08 (8)	K^i^—Zn—O2—Zn^v^	82.07 (6)
O1^iii^—K—Zn—O4	−95.13 (8)	O4—Zn—O2—K	−8.22 (8)
O6—K—Zn—O4	−137.47 (7)	O1—Zn—O2—K	113.32 (6)
O5^iv^—K—Zn—O4	−19.56 (8)	O2^v^—Zn—O2—K	−126.83 (7)
O6^iii^—K—Zn—O4	−53.81 (7)	Zn^v^—Zn—O2—K	−126.83 (7)
O2—K—Zn—O4	171.69 (8)	K^iii^—Zn—O2—K	77.02 (5)
B2^i^—K—Zn—O4	−23.73 (8)	K^i^—Zn—O2—K	−44.76 (6)
O3—K—Zn—O4	95.11 (7)	O4^i^—K—O2—B3	−168.31 (13)
B3—K—Zn—O4	−163.59 (8)	O3^ii^—K—O2—B3	−40.67 (14)
B2—K—Zn—O4	70.13 (8)	O1^iii^—K—O2—B3	11.21 (15)
O4^i^—K—Zn—O1	148.61 (6)	O6—K—O2—B3	18.64 (13)
O3^ii^—K—Zn—O1	−71.93 (8)	O6^iii^—K—O2—B3	87.72 (14)
O1^iii^—K—Zn—O1	3.02 (9)	O4—K—O2—B3	131.35 (14)
O6—K—Zn—O1	−39.32 (6)	B2^i^—K—O2—B3	114.45 (14)
O5^iv^—K—Zn—O1	78.59 (8)	O3—K—O2—B3	−140.57 (14)
O6^iii^—K—Zn—O1	44.34 (6)	B2—K—O2—B3	−158.37 (14)
O2—K—Zn—O1	−90.16 (8)	O4^i^—K—O2—Zn^v^	−29.68 (10)
O4—K—Zn—O1	98.15 (8)	O3^ii^—K—O2—Zn^v^	97.96 (9)
B2^i^—K—Zn—O1	74.42 (8)	O1^iii^—K—O2—Zn^v^	149.85 (8)
O3—K—Zn—O1	−166.75 (6)	O6—K—O2—Zn^v^	157.27 (11)
B3—K—Zn—O1	−65.44 (8)	O6^iii^—K—O2—Zn^v^	−133.65 (9)
B2—K—Zn—O1	168.27 (7)	O4—K—O2—Zn^v^	−90.02 (9)
O4^i^—K—Zn—O2^v^	−58.07 (6)	B2^i^—K—O2—Zn^v^	−106.92 (10)
O3^ii^—K—Zn—O2^v^	81.39 (8)	O3—K—O2—Zn^v^	−1.94 (7)
O1^iii^—K—Zn—O2^v^	156.34 (7)	B3—K—O2—Zn^v^	138.63 (18)
O6—K—Zn—O2^v^	114.00 (6)	B2—K—O2—Zn^v^	−19.73 (9)
O5^iv^—K—Zn—O2^v^	−128.09 (7)	O4^i^—K—O2—Zn	65.17 (7)
O6^iii^—K—Zn—O2^v^	−162.34 (6)	O3^ii^—K—O2—Zn	−167.19 (5)
O2—K—Zn—O2^v^	63.16 (9)	O1^iii^—K—O2—Zn	−115.30 (6)
O4—K—Zn—O2^v^	−108.53 (8)	O6—K—O2—Zn	−107.88 (8)
B2^i^—K—Zn—O2^v^	−132.26 (8)	O6^iii^—K—O2—Zn	−38.80 (5)
O3—K—Zn—O2^v^	−13.42 (6)	O4—K—O2—Zn	4.83 (5)
B3—K—Zn—O2^v^	87.88 (8)	B2^i^—K—O2—Zn	−12.07 (6)
B2—K—Zn—O2^v^	−38.41 (7)	O3—K—O2—Zn	92.91 (6)
O4^i^—K—Zn—O2	−121.23 (7)	B3—K—O2—Zn	−126.52 (15)
O3^ii^—K—Zn—O2	18.23 (7)	B2—K—O2—Zn	75.12 (7)
O1^iii^—K—Zn—O2	93.19 (8)	O4^i^—B2—O3—B1	179.7 (2)
O6—K—Zn—O2	50.85 (7)	O6^vi^—B2—O3—B1	−3.2 (3)
O5^iv^—K—Zn—O2	168.75 (8)	K^i^—B2—O3—B1	−83.4 (3)
O6^iii^—K—Zn—O2	134.50 (6)	K—B2—O3—B1	−162.96 (19)
O4—K—Zn—O2	−171.69 (8)	O4^i^—B2—O3—K^ii^	50.9 (3)
B2^i^—K—Zn—O2	164.58 (8)	O6^vi^—B2—O3—K^ii^	−132.00 (17)
O3—K—Zn—O2	−76.58 (7)	K^i^—B2—O3—K^ii^	147.80 (11)
B3—K—Zn—O2	24.72 (8)	K—B2—O3—K^ii^	68.26 (12)
B2—K—Zn—O2	−101.56 (8)	O4^i^—B2—O3—K	−17.4 (2)
O4^i^—K—Zn—Zn^v^	−89.18 (4)	O6^vi^—B2—O3—K	159.7 (2)
O3^ii^—K—Zn—Zn^v^	50.27 (6)	K^i^—B2—O3—K	79.54 (16)
O1^iii^—K—Zn—Zn^v^	125.23 (5)	O5^vi^—B1—O3—B2	7.7 (3)
O6—K—Zn—Zn^v^	82.89 (4)	O1^v^—B1—O3—B2	136.6 (2)
O5^iv^—K—Zn—Zn^v^	−159.20 (5)	O1^vii^—B1—O3—B2	−120.7 (2)
O6^iii^—K—Zn—Zn^v^	166.54 (4)	B1^viii^—B1—O3—B2	−171.5 (2)
O2—K—Zn—Zn^v^	32.04 (5)	K^ii^—B1—O3—B2	−140.0 (2)
O4—K—Zn—Zn^v^	−139.64 (6)	K^ix^—B1—O3—B2	74.7 (4)
B2^i^—K—Zn—Zn^v^	−163.37 (6)	O5^vi^—B1—O3—K^ii^	147.63 (16)
O3—K—Zn—Zn^v^	−44.54 (4)	O1^v^—B1—O3—K^ii^	−83.43 (18)
B3—K—Zn—Zn^v^	56.77 (6)	O1^vii^—B1—O3—K^ii^	19.24 (17)
B2—K—Zn—Zn^v^	−69.52 (6)	B1^viii^—B1—O3—K^ii^	−31.5 (3)
O4^i^—K—Zn—K^iii^	146.95 (4)	K^ix^—B1—O3—K^ii^	−145.3 (3)
O3^ii^—K—Zn—K^iii^	−73.59 (6)	O5^vi^—B1—O3—K	−137.1 (2)
O1^iii^—K—Zn—K^iii^	1.37 (4)	O1^v^—B1—O3—K	−8.2 (4)
O6—K—Zn—K^iii^	−40.97 (4)	O1^vii^—B1—O3—K	94.5 (3)
O5^iv^—K—Zn—K^iii^	76.93 (6)	B1^viii^—B1—O3—K	43.7 (4)
O6^iii^—K—Zn—K^iii^	42.68 (4)	K^ii^—B1—O3—K	75.3 (2)
O2—K—Zn—K^iii^	−91.82 (6)	K^ix^—B1—O3—K	−70.0 (4)
O4—K—Zn—K^iii^	96.49 (6)	O4^i^—K—O3—B2	10.06 (12)
B2^i^—K—Zn—K^iii^	72.76 (7)	O3^ii^—K—O3—B2	128.59 (13)
O3—K—Zn—K^iii^	−168.40 (3)	O1^iii^—K—O3—B2	98.98 (15)
B3—K—Zn—K^iii^	−67.10 (6)	O6—K—O3—B2	−150.28 (12)
B2—K—Zn—K^iii^	166.62 (5)	O5^iv^—K—O3—B2	42.17 (13)
O4^i^—K—Zn—K^i^	19.17 (4)	O6^iii^—K—O3—B2	−61.35 (14)
O3^ii^—K—Zn—K^i^	158.63 (6)	O2—K—O3—B2	−133.21 (13)
O1^iii^—K—Zn—K^i^	−126.42 (5)	O4—K—O3—B2	−63.66 (13)
O6—K—Zn—K^i^	−168.76 (4)	B2^i^—K—O3—B2	−51.73 (16)
O5^iv^—K—Zn—K^i^	−50.85 (5)	B3—K—O3—B2	−148.62 (13)
O6^iii^—K—Zn—K^i^	−85.10 (4)	O4^i^—K—O3—B1	161.0 (3)
O2—K—Zn—K^i^	140.40 (6)	O3^ii^—K—O3—B1	−80.5 (3)
O4—K—Zn—K^i^	−31.29 (6)	O1^iii^—K—O3—B1	−110.1 (3)
B2^i^—K—Zn—K^i^	−55.02 (6)	O6—K—O3—B1	0.7 (3)
O3—K—Zn—K^i^	63.81 (4)	O5^iv^—K—O3—B1	−166.9 (3)
B3—K—Zn—K^i^	165.12 (6)	O6^iii^—K—O3—B1	89.6 (3)
B2—K—Zn—K^i^	38.83 (5)	O2—K—O3—B1	17.7 (3)
O3^ii^—K—B2—O4^i^	101.89 (15)	O4—K—O3—B1	87.3 (3)
O1^iii^—K—B2—O4^i^	39.7 (2)	B2^i^—K—O3—B1	99.2 (3)
O6—K—B2—O4^i^	−158.69 (12)	B3—K—O3—B1	2.3 (3)
O5^iv^—K—B2—O4^i^	18.26 (15)	B2—K—O3—B1	150.9 (3)
O6^iii^—K—B2—O4^i^	−67.98 (15)	O4^i^—K—O3—K^ii^	−118.53 (7)
O2—K—B2—O4^i^	−155.45 (14)	O3^ii^—K—O3—K^ii^	0.0
O4—K—B2—O4^i^	−88.37 (15)	O1^iii^—K—O3—K^ii^	−29.61 (10)
B2^i^—K—B2—O4^i^	−69.11 (14)	O6—K—O3—K^ii^	81.13 (6)
O3—K—B2—O4^i^	160.9 (2)	O5^iv^—K—O3—K^ii^	−86.42 (6)
B3—K—B2—O4^i^	−165.01 (14)	O6^iii^—K—O3—K^ii^	170.06 (5)
O4^i^—K—B2—O3	−160.9 (2)	O2—K—O3—K^ii^	98.19 (5)
O3^ii^—K—B2—O3	−59.00 (14)	O4—K—O3—K^ii^	167.74 (5)
O1^iii^—K—B2—O3	−121.16 (12)	B2^i^—K—O3—K^ii^	179.68 (6)
O6—K—B2—O3	40.42 (16)	B3—K—O3—K^ii^	82.79 (6)
O5^iv^—K—B2—O3	−142.63 (12)	B2—K—O3—K^ii^	−128.59 (13)
O6^iii^—K—B2—O3	131.12 (12)	O1—Zn—O4—B2^i^	−37.9 (2)
O2—K—B2—O3	43.66 (12)	O2^v^—Zn—O4—B2^i^	−179.73 (18)
O4—K—B2—O3	110.73 (13)	O2—Zn—O4—B2^i^	81.2 (2)
B2^i^—K—B2—O3	129.99 (15)	Zn^v^—Zn—O4—B2^i^	129.37 (18)
B3—K—B2—O3	34.10 (14)	K—Zn—O4—B2^i^	73.35 (19)
O4^i^—K—B2—O6^vi^	74.8 (4)	K^iii^—Zn—O4—B2^i^	−1.03 (19)
O3^ii^—K—B2—O6^vi^	176.7 (4)	K^i^—Zn—O4—B2^i^	−177.6 (3)
O1^iii^—K—B2—O6^vi^	114.5 (4)	O1—Zn—O4—K^i^	139.70 (9)
O6—K—B2—O6^vi^	−83.9 (4)	O2^v^—Zn—O4—K^i^	−2.11 (12)
O5^iv^—K—B2—O6^vi^	93.0 (4)	O2—Zn—O4—K^i^	−101.18 (10)
O6^iii^—K—B2—O6^vi^	6.8 (4)	Zn^v^—Zn—O4—K^i^	−53.01 (11)
O2—K—B2—O6^vi^	−80.7 (4)	K—Zn—O4—K^i^	−109.03 (10)
O4—K—B2—O6^vi^	−13.6 (4)	K^iii^—Zn—O4—K^i^	176.59 (8)
B2^i^—K—B2—O6^vi^	5.7 (4)	O1—Zn—O4—K	−111.28 (6)
O3—K—B2—O6^vi^	−124.3 (5)	O2^v^—Zn—O4—K	106.92 (7)
B3—K—B2—O6^vi^	−90.2 (4)	O2—Zn—O4—K	7.84 (7)
O4^i^—K—B2—K^i^	69.11 (14)	Zn^v^—Zn—O4—K	56.02 (6)
O3^ii^—K—B2—K^i^	171.01 (5)	K^iii^—Zn—O4—K	−74.39 (5)
O1^iii^—K—B2—K^i^	108.85 (10)	K^i^—Zn—O4—K	109.03 (10)
O6—K—B2—K^i^	−89.57 (9)	O4^i^—K—O4—B2^i^	103.40 (14)
O5^iv^—K—B2—K^i^	87.38 (7)	O3^ii^—K—O4—B2^i^	−98.0 (2)
O6^iii^—K—B2—K^i^	1.13 (7)	O1^iii^—K—O4—B2^i^	−18.42 (14)
O2—K—B2—K^i^	−86.33 (7)	O6—K—O4—B2^i^	−92.60 (13)
O4—K—B2—K^i^	−19.26 (4)	O5^iv^—K—O4—B2^i^	36.17 (14)
B2^i^—K—B2—K^i^	0.0	O6^iii^—K—O4—B2^i^	−26.49 (12)
O3—K—B2—K^i^	−129.99 (15)	O2—K—O4—B2^i^	−135.79 (14)
B3—K—B2—K^i^	−95.89 (8)	O3—K—O4—B2^i^	151.02 (13)
O4^i^—K—B3—O2	15.32 (17)	B3—K—O4—B2^i^	−117.42 (14)
O3^ii^—K—B3—O2	139.97 (14)	B2—K—O4—B2^i^	127.65 (12)
O1^iii^—K—B3—O2	−170.63 (12)	O4^i^—K—O4—Zn	−126.01 (9)
O6—K—B3—O2	−146.5 (2)	O3^ii^—K—O4—Zn	32.6 (2)
O5^iv^—K—B3—O2	−163.33 (11)	O1^iii^—K—O4—Zn	112.17 (6)
O6^iii^—K—B3—O2	−91.29 (13)	O6—K—O4—Zn	37.99 (6)
O4—K—B3—O2	−44.29 (13)	O5^iv^—K—O4—Zn	166.76 (5)
B2^i^—K—B3—O2	−66.12 (14)	O6^iii^—K—O4—Zn	104.10 (8)
O3—K—B3—O2	36.98 (13)	O2—K—O4—Zn	−5.20 (5)
B2—K—B3—O2	23.39 (15)	B2^i^—K—O4—Zn	130.59 (15)
O4^i^—K—B3—O5	−97.2 (3)	O3—K—O4—Zn	−78.39 (6)
O3^ii^—K—B3—O5	27.5 (3)	B3—K—O4—Zn	13.17 (6)
O1^iii^—K—B3—O5	76.9 (3)	B2—K—O4—Zn	−101.76 (8)
O6—K—B3—O5	101.0 (3)	O4^i^—K—O4—K^i^	0.0
O5^iv^—K—B3—O5	84.2 (3)	O3^ii^—K—O4—K^i^	158.64 (19)
O6^iii^—K—B3—O5	156.2 (3)	O1^iii^—K—O4—K^i^	−121.82 (6)
O2—K—B3—O5	−112.5 (4)	O6—K—O4—K^i^	164.00 (6)
O4—K—B3—O5	−156.8 (3)	O5^iv^—K—O4—K^i^	−67.23 (7)
B2^i^—K—B3—O5	−178.6 (3)	O6^iii^—K—O4—K^i^	−129.90 (8)
O3—K—B3—O5	−75.5 (3)	O2—K—O4—K^i^	120.81 (7)
B2—K—B3—O5	−89.1 (3)	B2^i^—K—O4—K^i^	−103.40 (14)
O4^i^—K—B3—O6	161.80 (11)	O3—K—O4—K^i^	47.62 (5)
O3^ii^—K—B3—O6	−73.54 (13)	B3—K—O4—K^i^	139.18 (7)
O1^iii^—K—B3—O6	−24.14 (13)	B2—K—O4—K^i^	24.25 (6)
O5^iv^—K—B3—O6	−16.8 (2)	O2—B3—O5—B1^xi^	−171.9 (2)
O6^iii^—K—B3—O6	55.19 (13)	O6—B3—O5—B1^xi^	6.6 (3)
O2—K—B3—O6	146.5 (2)	K—B3—O5—B1^xi^	−76.4 (4)
O4—K—B3—O6	102.19 (13)	O2—B3—O5—K^xii^	40.2 (3)
B2^i^—K—B3—O6	80.36 (14)	O6—B3—O5—K^xii^	−141.28 (16)
O3—K—B3—O6	−176.54 (12)	K—B3—O5—K^xii^	135.7 (2)
B2—K—B3—O6	169.87 (12)	O2—B3—O6—B2^xi^	177.2 (2)
O4—Zn—O1—B1^v^	−173.34 (17)	O5—B3—O6—B2^xi^	−1.4 (3)
O2^v^—Zn—O1—B1^v^	−32.6 (2)	K—B3—O6—B2^xi^	142.9 (2)
O2—Zn—O1—B1^v^	59.85 (19)	O2—B3—O6—K	34.3 (2)
Zn^v^—Zn—O1—B1^v^	18.13 (19)	O5—B3—O6—K	−144.30 (19)
K—Zn—O1—B1^v^	119.73 (17)	O2—B3—O6—K^iii^	−76.7 (2)
K^iii^—Zn—O1—B1^v^	122.1 (2)	O5—B3—O6—K^iii^	104.7 (2)
K^i^—Zn—O1—B1^v^	−146.02 (16)	K—B3—O6—K^iii^	−111.02 (10)
O4—Zn—O1—B1^x^	−48.07 (17)	O4^i^—K—O6—B3	−35.0 (2)
O2^v^—Zn—O1—B1^x^	92.65 (16)	O3^ii^—K—O6—B3	101.87 (13)
O2—Zn—O1—B1^x^	−174.87 (15)	O1^iii^—K—O6—B3	155.58 (13)
Zn^v^—Zn—O1—B1^x^	143.40 (14)	O5^iv^—K—O6—B3	169.98 (12)
K—Zn—O1—B1^x^	−115.00 (15)	O6^iii^—K—O6—B3	−121.12 (14)
K^iii^—Zn—O1—B1^x^	−112.59 (17)	O2—K—O6—B3	−18.14 (12)
K^i^—Zn—O1—B1^x^	−20.75 (18)	O4—K—O6—B3	−76.92 (13)
O4—Zn—O1—K^iii^	64.52 (8)	B2^i^—K—O6—B3	−101.49 (14)
O2^v^—Zn—O1—K^iii^	−154.77 (6)	O3—K—O6—B3	3.86 (14)
O2—Zn—O1—K^iii^	−62.29 (8)	B2—K—O6—B3	−13.77 (16)
Zn^v^—Zn—O1—K^iii^	−104.01 (6)	O4^i^—K—O6—B2^xi^	−172.34 (18)
K—Zn—O1—K^iii^	−2.41 (8)	O3^ii^—K—O6—B2^xi^	−35.48 (19)
K^i^—Zn—O1—K^iii^	91.84 (7)	O1^iii^—K—O6—B2^xi^	18.23 (19)
O5—B3—O2—Zn^v^	6.8 (3)	O5^iv^—K—O6—B2^xi^	32.6 (2)
O6—B3—O2—Zn^v^	−171.72 (14)	O6^iii^—K—O6—B2^xi^	101.5 (2)
K—B3—O2—Zn^v^	−138.72 (16)	O2—K—O6—B2^xi^	−155.5 (2)
O5—B3—O2—Zn	−127.1 (2)	O4—K—O6—B2^xi^	145.74 (19)
O6—B3—O2—Zn	54.3 (3)	B2^i^—K—O6—B2^xi^	121.17 (17)
K—B3—O2—Zn	87.33 (13)	O3—K—O6—B2^xi^	−133.48 (18)
O5—B3—O2—K	145.6 (2)	B3—K—O6—B2^xi^	−137.3 (3)
O6—B3—O2—K	−33.0 (2)	B2—K—O6—B2^xi^	−151.1 (2)
O4—Zn—O2—B3	−96.81 (18)	O4^i^—K—O6—K^iii^	86.13 (14)
O1—Zn—O2—B3	24.73 (18)	O3^ii^—K—O6—K^iii^	−137.01 (6)
O2^v^—Zn—O2—B3	144.6 (2)	O1^iii^—K—O6—K^iii^	−83.30 (6)
Zn^v^—Zn—O2—B3	144.6 (2)	O5^iv^—K—O6—K^iii^	−68.90 (8)
K—Zn—O2—B3	−88.59 (17)	O6^iii^—K—O6—K^iii^	0.0
K^iii^—Zn—O2—B3	−11.57 (16)	O2—K—O6—K^iii^	102.98 (8)
K^i^—Zn—O2—B3	−133.34 (16)	O4—K—O6—K^iii^	44.20 (5)
O4—Zn—O2—Zn^v^	118.60 (7)	B2^i^—K—O6—K^iii^	19.63 (6)
O1—Zn—O2—Zn^v^	−119.85 (7)	O3—K—O6—K^iii^	124.98 (6)
O2^v^—Zn—O2—Zn^v^	0.0	B3—K—O6—K^iii^	121.12 (14)
K—Zn—O2—Zn^v^	126.83 (7)	B2—K—O6—K^iii^	107.35 (9)

Symmetry codes: (i) −*x*+1, −*y*+1, −*z*+1; (ii) −*x*, −*y*+1, −*z*+1; (iii) −*x*+1, −*y*, −*z*+1; (iv) *x*, *y*, *z*+1; (v) −*x*+1, −*y*+1, −*z*; (vi) *x*, *y*+1, *z*; (vii) *x*−1, *y*+1, *z*; (viii) −*x*, −*y*+2, −*z*; (ix) *x*, *y*+1, *z*−1; (x) *x*+1, *y*−1, *z*; (xi) *x*, *y*−1, *z*; (xii) *x*, *y*, *z*−1.
